# Disparities in Physical Activity and Sport Participation Among Transition‐Age Youth With Autism and Intellectual Disability

**DOI:** 10.1002/aur.70277

**Published:** 2026-05-13

**Authors:** Daniel E. Lidstone

**Affiliations:** ^1^ School of Behavioral Sciences and Education, Penn State Harrisburg Middletown Pennsylvania USA

**Keywords:** adolescence, autism spectrum disorder (ASD), health equity, intellectual disability (ID), physical activity, public health, socioeconomic disparities

## Abstract

Physical activity is critical for health, yet many adolescents do not engage in regular activity. Youth with autism spectrum disorder (ASD) and intellectual disability (ID) may be at heightened risk due to motor, sensory, and social barriers, but less is known about complete disengagement. Using nationally representative data from the 2021–2023 National Survey of Children's Health, this study examined disparities in (1) any weekly physical activity and (2) participation in organized sport among transition‐age youth (14–17 years) with ASD only, ID only, or ASD + ID, compared with youth without ASD or ID. Survey‐weighted multiple logistic regression models estimated adjusted odds ratios (aORs) for each outcome, controlling for sex, race, household poverty ratio, metropolitan residence, and age. Compared to youth without ASD or ID, all diagnostic groups had significantly lower odds of engaging in any weekly physical activity (ASD only: aOR = 0.32, 95% CI: 0.24–0.44; ID only: aOR = 0.49, 95% CI: 0.31–0.76; ASD + ID: aOR = 0.51, 95% CI: 0.30–0.88; all *p* ≤ 0.02) and organized sport (ASD only: aOR = 0.20, 95% CI: 0.15–0.27; ID only: aOR = 0.31, 95% CI: 0.19–0.51; ASD + ID: aOR = 0.24, 95% CI: 0.14–0.41; all *p* < 0.001). Across both models and diagnostic groups, female sex, lower household income, and increased age were associated with lower physical activity and sport participation (all *p* ≤ 0.02), whereas non‐metropolitan residence was associated with higher odds of sport participation‐only (*p* = 0.005). These findings identify substantial disengagement among youth with ASD and/or ID and highlight the need for accessible, adapted physical activity and sport opportunities during adolescence to reduce risk of long‐term inactivity.

## Summary

Adolescents with autism and/or intellectual disability are less likely to take part in weekly physical activity and sports than their peers without these conditions. Lower engagement was most pronounced for autistic youth without intellectual disability. The findings point to a need for more inclusive and sustainable physical activity and organized sport opportunities before support services decline in adulthood.

## 1 Introduction

Physical inactivity in childhood is a major public health concern, with fewer than one in four U.S. children achieving the recommended 60 min of daily moderate‐to‐vigorous physical activity (U.S. Department of Health and Human Services 2018). Insufficient physical activity increases risk for obesity, cardiometabolic disease, depression, and poorer academic outcomes (Hillman et al. 2008). These risks are particularly amplified for youth with autism spectrum disorder (ASD) and intellectual disability (ID), who encounter multi‐level barriers to participation including motor difficulties, sensory sensitivities, stigma, and limited access to developmentally appropriate physical activity opportunities (Emerson 2005; O'Flaherty et al. 2025; Pan and Frey 2006; Wong et al. 2024).

Prior work has largely focused on failure to meet physical activity guidelines (Gehricke et al. 2020; McCoy et al. 2016; Schibler et al. 2025; Stanish et al. 2017; Tyler et al. 2014), with less attention to youth who are completely disengaged from physical activity or organized sport. Identifying transition‐aged youth with ASD and/or ID at risk for complete inactivity and no sport participation is critical to prevent inactivity in adulthood when access to structured programs declines after adolescence (Weiss et al. 2021). This transition is further complicated by loss of services following high school, particularly for youth with ASD and co‐occurring ID (Laxman et al. 2019), which may reduce opportunities for physical activity and negatively impact physical and social well‐being (Biggs and Carter 2016). Early and sustained engagement in physical activity and sport is therefore important not only for physical health, but also for supporting independence, social participation, and quality of life. Evidence further suggests that physical activity and sport may mitigate depression and enhance cognitive functioning (Grosprêtre et al. 2024; Hedley et al. 2017; Lloyd et al. 2023). Despite these concerns, existing research has largely treated youth with ASD and ID as homogeneous groups, limiting understanding of how disengagement from physical activity and sport varies across clinically distinct subgroups.

Few studies have examined disengagement from physical activity and sport across clinically distinct neurodevelopmental subgroups, including autistic youth with and without co‐occurring ID, or compared these patterns to youth with ID alone. The current study addresses this gap by modeling diagnosis as a four‐level categorical variable (no ASD or ID [reference], ASD only, ID only, ASD + ID), enabling direct comparison of participation patterns across groups.

This brief report uses nationally representative data from the 2021–2023 National Survey of Children's Health (NSCH) survey to examine disparities in (1) weekly physical activity and (2) sport participation among transition‐age youth (14–17 years) with ASD and/or ID compared to youth without these conditions.

## 2 Methods

### 2.1 Data Source

Data were drawn from the 2021–2023 National Survey of Children's Health (NSCH), a cross‐sectional survey administered annually by the U.S. Census Bureau on behalf of the Health Resources and Services Administration (https://www.census.gov/programs‐surveys/nsch/data/datasets.html). The NSCH provides nationally representative estimates of the physical and emotional health of children and youth ages 0–17 years in the United States. Data are collected through mailed invitations and online surveys completed by parents or guardians. Because the NSCH is a publicly available, de‐identified dataset, this study did not require Institutional Review Board (IRB) approval.

### 2.2 Study Sample

The analytic sample included transition‐age youth (ages 14–17 years) with complete data on physical activity, diagnostic status, and covariates. Participants were excluded if they were outside the age range or had missing data on physical activity, sports participation, ASD/ID diagnosis, sex, race, federal poverty ratio, or metro status. After exclusions, the final analytic sample was 26,308 youth, of whom 25,024 had no ASD or ID, 868 had ASD without ID (ASD only), 221 had ID without ASD (ID only), and 195 had both ASD and ID (ASD + ID).

### 2.3 Measures

Physical activity (*Outcome 1*) was assessed by parent report of the number of days in the past week the child engaged in ≥ 60 min of activity and was dichotomized as no activity versus any activity (≥ 1 day/week). Sport participation (*Outcome 2*) was defined as parent‐reported participation in a sports team or lessons outside of school hours in the past 12 months (yes/no).

Diagnosis was categorized into four groups: no ASD or ID, ASD only, ID only, and ASD + ID. Classification of diagnosis required parent report that a healthcare provider had diagnosed the condition and that the child currently had the condition.

Sociodemographic covariates included sex (male/female), race (White vs. non‐White), household federal poverty ratio (≤ 200% vs. > 200%), metropolitan residence (metropolitan vs. non‐metropolitan), and age (14 vs. 15, 16, 17 years). Additional details on NSCH‐derived variables are provided in the Supplemental File.

No written consent has been obtained from the patients as there is no patient‐identifiable data included.

### 2.4 Statistical Analysis

Descriptive statistics were used to compare youth with and without weekly physical activity across diagnosis and sociodemographic characteristics. All estimates accounted for the complex sampling design of the National Survey of Children's Health (NSCH), including stratification (state, sampling strata), clustering (household), and three‐year averaged sampling weights (survey weight divided by three) using the *survey* package in R. Bivariate associations between demographic and diagnostic variables and both physical activity and sport participation status were assessed using Rao‐Scott adjusted chi‐square tests to account for the complex survey design.

Separate survey‐weighted multiple logistic regression models were used to estimate adjusted odds ratios (aORs) and 95% confidence intervals (CIs) for engaging in any physical activity (*model 1*) and participation in sports (*model 2*), with diagnosis group (no ASD or ID [reference], ASD only, ID only, ASD + ID) as the primary independent variable. Sociodemographic covariates for the multiple logistic regression models included sex (male [reference] vs. female), race (White [reference] vs. non‐White), household poverty ratio (> 200% [reference] vs. ≤ 200% of the federal poverty level), metropolitan status (metropolitan [reference] vs. non‐metropolitan), and age (14 [reference] vs. 15, 16, and 17 years). Models were fit using a quasibinomial distribution to account for the survey design. The overall significance of the adjusted models was assessed using design‐based Wald tests comparing the full model to an intercept‐only model.

Only non‐imputed variables were used for poverty ratio, race, and sex, and cases with missing data were excluded. Statistical significance was defined as *p* < 0.05. All analyses were conducted in *R* (version 2024.04.2) using the *survey* package.

## 3 Results

### 3.1 Proportion of Any Weekly Physical Activity and Any Annual Sport Participation

**FIGURE 1:**
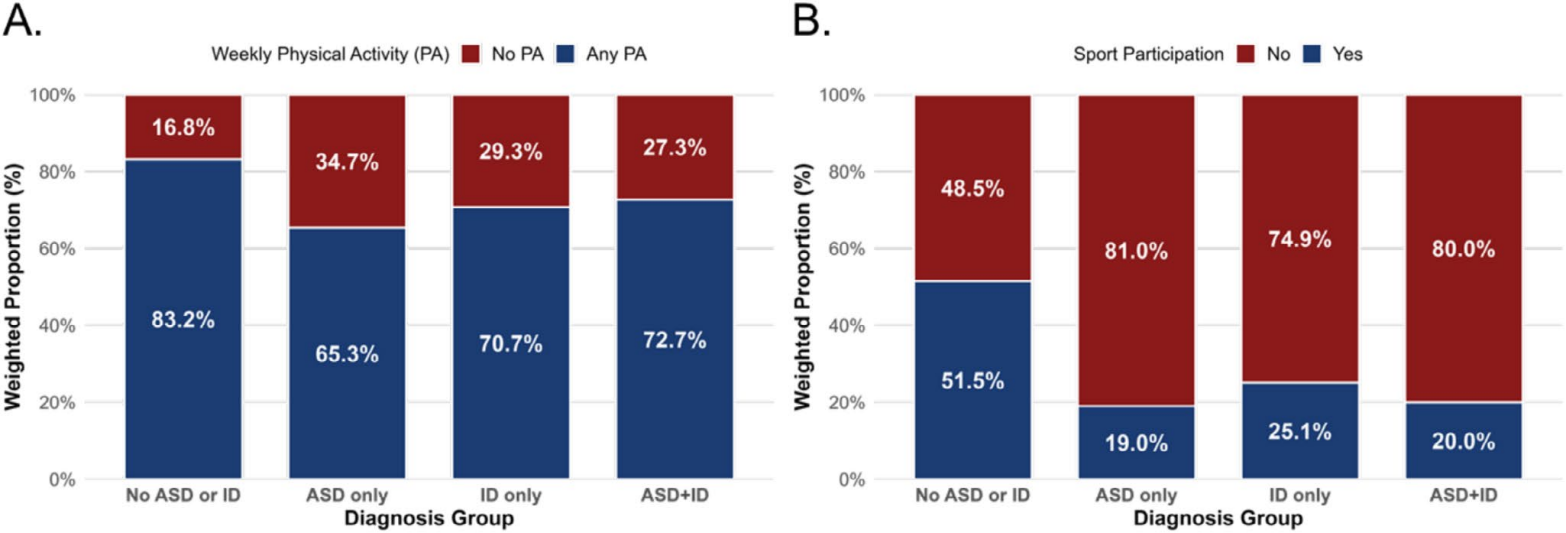
(A) Physical activity and (B) sport participation status by diagnosis group among U.S. youth aged 14–17 years. (A—*left*): Stacked bars show survey‐weighted proportions of youth reporting no physical activity (red/top) and any physical activity (blue/bottom) within each diagnosis group. (B—*right*): Stacked bars show survey‐weighted proportions of youth reporting no sport participation (red/top) versus any sport participation (blue/bottom) within each diagnosis group. Percentages are weighted to reflect the U.S. population. ASD, autism spectrum disorder; ID, intellectual disability; PA, physical activity.

Among the 26,308 youth aged 14–17 years in the analytic sample, 16.8% of those without ASD or ID reported no weekly PA (≤ 60 min), compared with 34.7% of youth with ASD only, 29.3% with ID only, and 27.3% with ASD + ID. For sports participation, 48.5% of those without ASD or ID reported no sport participation in the past 12 months, compared with 81% of youth with ASD only, 74.9% with ID only, and 80% with ASD + ID (Table 1, Figure 1).

**TABLE 1:** Weighted prevalence of physical activity and sport participation by diagnosis among U.S. youth aged 14–17 years.

Diagnosis	Total (*n*/weighted *n*)	Physical activity		Sport participation	
		No PA *n*/weighted *n* (%)	Any PA *n*/weighted *n* (%)	No sport *n*/weighted *n* (%)	Yes sport *n*/weighted *n* (%)
No ASD or ID	25,024/11,394,819	3626/1,912,955 (16.8%)	21,398/9,481,864 (83.2%)	11,139/5,527,976 (48.5%)	13,885/5,866,843 (51.5%)
ASD only	868/331,385	289/114,834 (34.7%)	579/216,551 (65.3%)	685/268,341 (81.0%)	183/63,044 (19.0%)
ID only	221/86,506	67/25,306 (29.3%)	154/61,200 (70.7%)	154/64,791 (74.9%)	67/21,715 (25.1%)
ASD + ID	195/88,739	60/24,238 (27.3%)	135/64,501 (72.7%)	155/70,993 (80.0%)	40/17,746 (20.0%)

*Note*: Values are reported as unweighted counts and survey‐weighted population estimates (*n*/weighted *n*), with weighted row percentages. Percentages represent the proportion of youth within each diagnosis group reporting no participation (physical activity, sport) or any participation (physical activity, sport).

Abbreviations: ASD, autism spectrum disorder; ID, intellectual disability; PA, physical activity.

Bivariate associations between diagnosis and sociodemographic variables for physical activity and sport participation are presented in the supplemental material (Tables S1 and S3). Rao‐Scott adjusted chi‐square tests indicated that both physical activity and sport participation status differed significantly by diagnosis (physical activity: Rao‐Scott *χ* ^2^ = 25.35, *p* < 0.001; sport participation: Rao‐Scott *χ* ^2^ = 57.77, p < 0.001) and sociodemographic factors (race, poverty ratio, sex, and age; all *p* < 0.03), but not by metropolitan residence (*p* > 0.05).

### 3.2 Main Logistical Regression Results for Any Weekly Physical Activity and Any Sport Participation

In survey‐weighted logistic regression models, both adjusted models were statistically significant (physical activity: *F* = 18.98, *p* < 0.001; sport participation: *F* = 36.74, *p* < 0.001).

#### 3.2.1 Effect of Diagnosis on Physical Activity and Sport Participation

**FIGURE 2:**
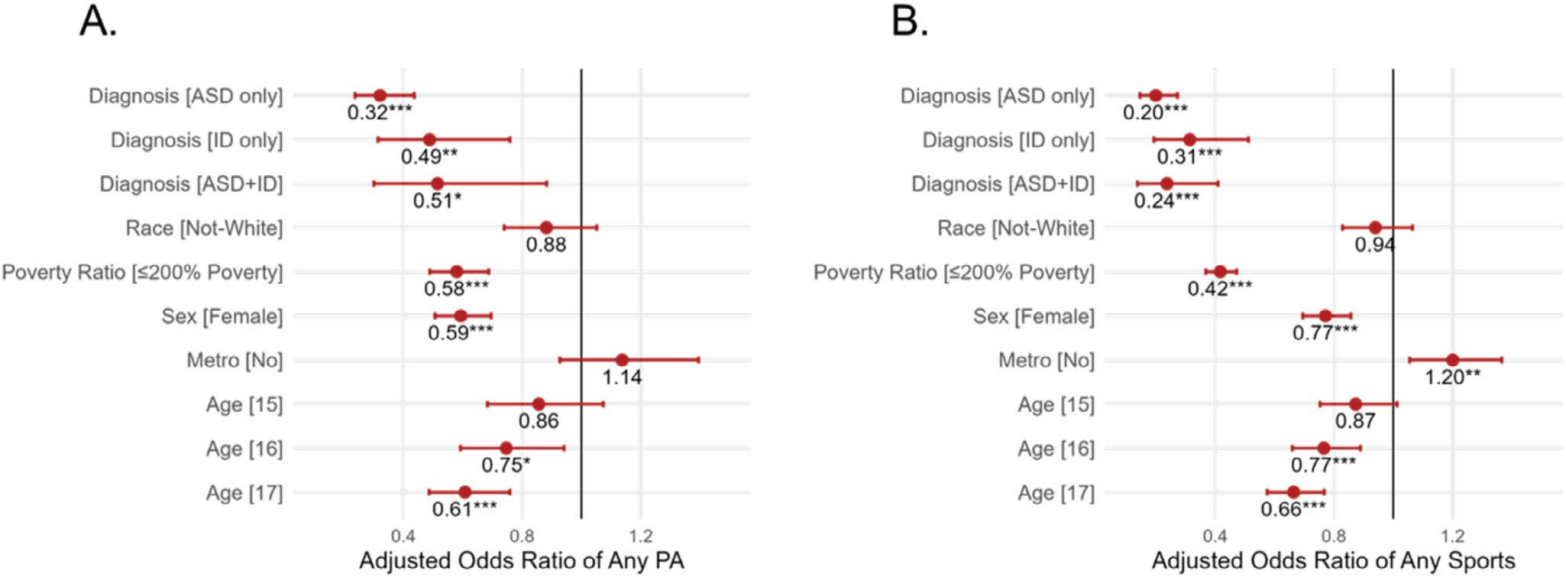
Adjusted odds ratios (aORs) for engagement in (A) Any physical activity and (B) Any sport participation among U.S. youth aged 14–17 years. Points represent adjusted odds ratios (aORs) and horizontal lines indicate 95% confidence intervals from survey‐weighted logistic regression models accounting for the complex sampling design of the National Survey of Children's Health. The vertical reference line at 1.0 indicates no association. Models were adjusted for sex, race, household poverty ratio, metropolitan residence, and age. (*p < 0.05, **p < 0.01, ***p < 0.001). ASD, autism spectrum disorder; ID, intellectual disability; PA, physical activity.

Diagnosis was consistently associated with lower odds of engagement across outcomes. For weekly physical activity, youth with ASD only (aOR = 0.32, 95% CI: 0.24–0.44, *p* < 0.001), ID only (aOR = 0.49, 95% CI: 0.31–0.76, *p* = 0.001), and ASD + ID (aOR = 0.51, 95% CI: 0.30–0.88, *p* = 0.016) had reduced odds compared with youth without these conditions. Similar but more pronounced reductions were observed for sport participation (ASD only: aOR = 0.20, 95% CI: 0.15–0.27, *p* < 0.001; ID only: aOR = 0.31, 95% CI: 0.19–0.51, *p* < 0.001; ASD + ID: aOR = 0.24, 95% CI: 0.14–0.41, *p* < 0.001) (Figure 2, Tables S2 and S4).

#### 3.2.2 Effect of Sociodemographic Covariates on Physical Activity and Sport Participation Across Diagnostic Groups

Lower socioeconomic status (≤ 200% federal poverty level), female sex, and increased age (ages 16 and 17 years) were associated with lower odds of both weekly physical activity (poverty: aOR = 0.59, 95% CI: 0.49–0.70, *p* < 0.001; female: aOR = 0.59, 95% CI: 0.51–0.70, *p* < 0.001; age [16]: aOR = 0.75, 95% CI: 0.59–0.94, *p* = 0.01; age [17]: aOR = 0.61, 95% CI: 0.49–0.76, *p* < 0.001) and sport participation (poverty: aOR = 0.42, 95% CI: 0.37–0.48, *p* < 0.001; female: aOR = 0.77, 95% CI: 0.70–0.86, *p* < 0.001; age [16]: aOR = 0.77, 95% CI: 0.66–0.89, *p* < 0.001; age [17]: aOR = 0.66, 95% CI: 0.57–0.77, *p* < 0.001). Race was not associated with either outcome after adjustment (*p* > 0.16). Non‐metropolitan residence was not associated with weekly physical activity (*p* = 0.21) but was associated with higher odds of sport participation (aOR = 1.20, 95% CI: 1.06–1.37, *p* = 0.005).

## 4 Discussion

This brief report examined disparities in physical activity and sport participation among transition‐age youth (14–17 years) with ASD and/or ID compared with youth without these conditions. Across both outcomes, youth with ASD and/or ID had significantly lower odds of engagement, with the largest disparities observed for sport participation. These findings highlight the potential to increase engagement in physical activity through increased access and participation in sports among autistic youth, including those without co‐occurring ID.

A central contribution of this study is the focus on complete inactivity, which identifies a meaningful subgroup of youth with ASD and/or ID who are entirely disengaged from general physical activity and organized sport. This distinction has practical implications, as complete inactivity likely reflects the accumulation of multiple, interacting barriers across individual, social, and environmental domains, rather than simply lower levels of engagement (Arkesteyn et al. 2025; Must et al. 2015). In contrast, youth with low but non‐zero participation may face more circumscribed or modifiable barriers. Accordingly, these groups may require fundamentally different intervention approaches, with completely inactive youth requiring strategies focused on access, initiation, and barrier reduction, and partially engaged youth benefiting from approaches that enhance skill, enjoyment, and sustained participation. Addressing barriers that contribute to complete disengagement may be particularly important for mitigating elevated risk for adverse physical and mental health outcomes in adulthood (Sedgewick et al. 2020; Ward et al. 2023), and for improving the effectiveness of community‐based programs that aim to increase participation rather than initiate it.

Autistic youth without intellectual disability (ASD only) demonstrated the lowest odds of engagement relative to peers without ASD or ID, highlighting a need for targeted programming for this subgroup. Youth with intellectual disability may have greater access to structured supports (e.g., adapted school‐based programming and disability‐specific sport opportunities such as Special Olympics), whereas more cognitively able autistic youth are more likely to participate in integrated physical education and sport alongside neurotypical peers. In these integrated contexts, motor and social differences may be more visible and associated with negative experiences, including bullying and social isolation (Jachyra et al. 2021; Okkenhaug et al. 2024; Waddington et al. 2025). Early negative experiences in physical education, combined with persistent social isolation into adulthood (Orsmond et al. 2013) may contribute to long‐term disengagement from physical activity and organized sport. In the absence of adapted opportunities tailored to autistic youth without intellectual disability, this group may lack a clear sense of belonging in either disability‐specific or integrated settings, in part due to misperceptions about the nature and severity of their needs and limited experience among physical educators and coaches in working with autistic youth (Skrubbeltrang et al. 2026). Future research should evaluate interventions designed to improve physical activity participation and enjoyment among autistic youth across the cognitive spectrum, with particular attention to more cognitively able autistic youth. Given the observed decline in participation during the transition out of high school (ages 16–17), this developmental window may represent a critical period for intervention.

This study has several limitations. All measures were parent‐reported, including diagnosis, physical activity, and sport participation, and may therefore be subject to recall or reporting bias. The cross‐sectional design does not allow conclusions about temporal or directional relationships. Dichotomizing weekly physical activity as no activity versus any activity does not capture guideline adherence and may obscure meaningful differences in frequency. In addition, the NSCH does not assess activity intensity, duration beyond 60 min, activity context, or the frequency and type of sport participation. It also lacks detailed information on environmental and family‐level barriers, such as program availability, accessibility, cost, transportation, and social support, and may underrepresent families with the greatest disability‐related burden.

In conclusion, disparities in physical activity and sport participation among transition‐age youth with ASD and/or ID are a major public health concern placing this population at elevated risk for adverse health outcomes in adulthood. Policy efforts should prioritize increasing access to adapted, community‐based sport and physical activity programs that support regular engagement in weekly physical activity and provide structured opportunities for peer interaction. Early, inclusive, and sustainable strategies that emphasize autonomy are needed to facilitate initiation and maintenance of physical activity into adulthood. Coordinated efforts across schools, families, healthcare systems, and community organizations will be critical to support long‐term health behaviors in this population.

## Funding

The author has nothing to report.

## Ethics Statement

This research used de‐identified, publicly available data from the National Survey of Children's Health (NSCH) and was exempt from IRB approval.

## Consent

This study used de‐identified, publicly available data from the National Survey of Children's Health (NSCH: 2021–2023).

## Conflicts of Interest

The author declares no conflicts of interest.

## Supporting information


**Table S1:** Sample characteristics and bivariate associations between demographic and diagnostic variables and weekly physical activity status among U.S. youth aged 14–17 years. Values are presented as unweighted *n*/weighted *n* (weighted column %). Estimates account for the complex sampling design of the National Survey of Children's Health, including stratification, clustering, and sampling weights. Group differences were assessed using Rao–Scott adjusted chi‐square tests.
**Table S2:**: Crude and adjusted odds ratios for any physical activity. Estimates are from survey‐weighted logistic regression models accounting for complex survey design. Adjusted models include diagnosis, sex, race, poverty ratio, metropolitan status, and age. Reference groups are indicated.
**Table S3:** Sample characteristics and bivariate associations between demographic and diagnostic variables and sport participation status in the past 12 months among U.S. youth aged 14–17 years. Values are presented as unweighted *n*/weighted *n* (weighted column %). Estimates account for the complex sampling design of the National Survey of Children's Health, including stratification, clustering, and sampling weights. Group differences were assessed using Rao–Scott adjusted chi‐square tests.
**Table S4:** Crude and adjusted odds ratios for any sport participation in the past 12 months. Estimates are from survey‐weighted logistic regression models accounting for complex survey design. Adjusted models include diagnosis, sex, race, poverty ratio, metropolitan status, and age. Reference groups are indicated.

## Data Availability

The datasets analyzed in this study (National Survey of Children's Health 2021–2023) are available online on the US Census Bureau Website: https://www.census.gov/programs‐surveys/nsch/data/datasets.html.
