# Supplementary material for: Disparities in Physical Activity and Sport Participation Among Transition‐Age Youth With Autism and Intellectual Disability
Source: Autism Res. 2026 May 13;19(6):e70277. doi: 10.1002/aur.70277 (PMC13276677; doi:10.1002/aur.70277)
Supplement: Supplementary file 1 — Table S1: Sample characteristics and bivariate associations between demographic and diagnostic variables and weekly physical activity status among U.S. youth aged 14–17 years. Values are presented as unweighted n/weighted n (weighted column %). Estimates account for the complex sampling design of the National Survey of Children's Health, including stratification, clustering, and sampling weights. Group differences were assessed using Rao–Scott adjusted chi‐square tests. Table S2:: Crude and adjusted odds ratios for any physical activity. Estimates are from survey‐weighted logistic regression models accounting for complex survey design. Adjusted models include diagnosis, sex, race, poverty ratio, metropolitan status, and age. Reference groups are indicated. Table S3: Sample characteristics and bivariate associations between demographic and diagnostic variables and sport participation status in the past 12 months among U.S. youth aged 14–17 years. Values are presented as unweighted n/weighted n (weighted column %). Estimates account for the complex sampling design of the National Survey of Children's Health, including stratification, clustering, and sampling weights. Group differences were assessed using Rao–Scott adjusted chi‐square tests. Table S4: Crude and adjusted odds ratios for any sport participation in the past 12 months. Estimates are from survey‐weighted logistic regression models accounting for complex survey design. Adjusted models include diagnosis, sex, race, poverty ratio, metropolitan status, and age. Reference groups are indicated. [file AUR-19-0-s001.docx]

**SUPPLEMENT**

**Variable Coding**

**Physical Activity (PHYSACTIV)**

- Item: *“During the past week, on how many days did this child exercise, play a sport, or participate in physical activity for at least 60 minutes?”*
- Response options:
  - 1 = 0 days
  - 2 = 1–3 days
  - 3 = 4–6 days
  - 4 = Every day
- Recoded for analysis: *No PA (0 days)* vs. *Any PA (≥1 day)*.

**Sports Team or Sports Lessons - Past 12 Months (K7Q30)**

- Item: *“During the past 12 months, did this child participate in: A sports team or did they take sports lessons after school or on weekends?”*
  - 1 = Yes
  - 2 = No

**Autism Spectrum Disorder (ASD)**

- Derived from two questions:
  - (A) “Has a doctor or other health care provider ever told you that this child has autism?”
  - (B) “Does this child currently have autism?”
- Both A and B had to be answered “Yes” for the child to be classified as having ASD.
- Severity: Parents rated the child’s autism as mild, moderate, or severe.

**Intellectual Disability (ID)**

- Derived from two questions:
  - (A) *“Has a doctor or other health care provider ever told you that this child has intellectual disability?”*
  - (B) *“Does this child currently have intellectual disability?”*
- Both A and B had to be answered “Yes” for the child to be classified as having ID.
- Severity: Parents rated the child’s intellectual disability as *mild, moderate, or severe*.

**Race (SC_RACE_R)**

- Item: *“What is this child’s race?”*
- Response options:
  - 1 = White alone
  - 2 = Black or African American alone
  - 3 = American Indian or Alaska Native alone
  - 4 = Asian alone
  - 5 = Native Hawaiian and Other Pacific Islander alone
  - 7 = Two or More Races
- Recoded for analysis: *White* (reference) vs. *Not White* (categories 2–5, 7 combined).

**Sex**

- Parent-reported sex of the child:
  - 1 = Male
  - 2 = Female

**Poverty Ratio (FPL_R)**

- Based on reported household income relative to the federal poverty line.
- Recoded as: ≤*200% of FPL* vs. *>200% of FPL*.

**Metro Status (METRO_YN)**

- Variable: *Metropolitan Statistical Area Status (Operational)*
- Coding:
  - 1 = Metropolitan Statistical Area
  - 2 = Not Metropolitan Statistical Area
- Recoded for analysis: *Yes (Metropolitan)* vs. *No (Not Metropolitan)*.

**Physical Activity Participation**

1. **Sample characteristics and bivariate associations between demographic and diagnostic variables and weekly physical activity status.**

**Table S1. Sample characteristics and bivariate associations between demographic and diagnostic variables and weekly physical activity status among U.S. youth aged 14–17 years.** Values are presented as unweighted n / weighted n (weighted column %). Estimates account for the complex sampling design of the National Survey of Children’s Health, including stratification, clustering, and sampling weights. Group differences were assessed using Rao–Scott adjusted chi-square tests.

|  | **All (N=26,308) n / weighted n (%)** | **No PA (N=4,042) n / weighted n (%)** | **Any PA (N=22,266) n / weighted n (%)** | **Rao-Scott χ²; p-value** |
| --- | --- | --- | --- | --- |
| **Diagnosis:** |  |  |  |  |
| No ASD or ID | 25,024 / 11,394,819 (95.7%) | 3,626 / 1,912,955 (92.1%) | 21,398 / 9,481,864 (96.5%) | **χ² = 25.35; p = <0.001** |
| ASD only | 868 / 331,385 (2.8%) | 289 / 114,834 (5.5%) | 579 / 216,551 (2.2%) |  |
| ID only | 221 / 86,506 (0.7%) | 67 / 25,306 (1.2%) | 154 / 61,200 (0.6%) |  |
| ASD+ID | 195 / 88,739 (0.7%) | 60 / 24,238 (1.2%) | 135 / 64,501 (0.7%) |  |
| **Race:** |  |  |  |  |
| White | 20,318 / 8,455,577 (71.0%) | 2,979 / 1,406,965 (67.7%) | 17,339 / 7,048,612 (71.7%) | **χ² = 4.83; p = 0.028** |
| Not-White | 5,990 / 3,445,872 (29.0%) | 1,063 / 670,368 (32.3%) | 4,927 / 2,775,504 (28.3%) |  |
| **Poverty Ratio:** |  |  |  |  |
| >200% Poverty | 19,725 / 7,798,982 (65.5%) | 2,721 / 1,135,410 (54.7%) | 17,004 / 6,663,572 (67.8%) | **χ² = 45.50; p = <0.001** |
| ≤200% Poverty | 6,583 / 4,102,468 (34.5%) | 1,321 / 941,923 (45.3%) | 5,262 / 3,160,544 (32.2%) |  |
| **Sex:** |  |  |  |  |
| Male | 13,542 / 5,987,230 (50.3%) | 1,715 / 842,618 (40.6%) | 11,827 / 5,144,612 (52.4%) | **χ² = 36.49; p = <0.001** |
| Female | 12,766 / 5,914,219 (49.7%) | 2,327 / 1,234,715 (59.4%) | 10,439 / 4,679,504 (47.6%) |  |
| **Metropolitan Residence:** |  |  |  |  |
| Yes | 21,393 / 10,368,789 (87.1%) | 3,325 / 1,828,497 (88.0%) | 18,068 / 8,540,292 (86.9%) | χ² = 1.01; p = 0.315 |
| No | 4,915 / 1,532,661 (12.9%) | 717 / 248,837 (12.0%) | 4,198 / 1,283,824 (13.1%) |  |
| **Age (years):** |  |  |  |  |
| 14 | 5,862 / 3,072,471 (25.8%) | 756 / 443,526 (21.4%) | 5,106 / 2,628,945 (26.8%) | **χ² = 6.45; p = <0.001** |
| 15 | 6,427 / 3,027,145 (25.4%) | 952 / 494,396 (23.8%) | 5,475 / 2,532,749 (25.8%) |  |
| 16 | 6,964 / 2,947,343 (24.8%) | 1,077 / 532,603 (25.6%) | 5,887 / 2,414,741 (24.6%) |  |
| 17 | 7,055 / 2,854,490 (24.0%) | 1,257 / 606,808 (29.2%) | 5,798 / 2,247,682 (22.9%) |  |

1. **Crude and adjusted odds ratios for any physical activity**

**Table S2: Crude and adjusted odds ratios for any physical activity.** Estimates are from survey-weighted logistic regression models accounting for complex survey design. Adjusted models include diagnosis, sex, race, poverty ratio, metropolitan status, and age. Reference groups are indicated.

|  | **Crude OR**  **(95% CI)**  **p-value** | **aOR**  **(95% CI)**  **p-value** |
| --- | --- | --- |
| **Diagnosis:** |  |  |
| No ASD or ID | *Ref* | *Ref* |
| ASD only | **0.38 (0.29–0.50)**  **p = <0.001** | **0.32 (0.24–0.44)**  **p < 0.001** |
| ID only | **0.49 (0.32–0.76)**  **p = 0.001** | **0.49 (0.31–0.76)**  **p = 0.001** |
| ASD+ID | **0.54 (0.33–0.87)**  **p = 0.012** | **0.51 (0.30–0.88)**  **p = 0.016** |
| **Race:** |  |  |
| White | *Ref* | *Ref* |
| Not-White | **0.83 (0.70–0.98)**  **p = 0.028** | 0.88 (0.74**–**1.05)  p = 0.162 |
| **Poverty Ratio:** |  |  |
| >200% Poverty | *Ref* | *Ref* |
| ≤200% Poverty | **0.57 (0.49–0.67)**  **p < 0.001** | **0.58 (0.49–0.69)**  **p < 0.001** |
| **Sex:** |  |  |
| Male | *Ref* | *Ref* |
| Female | **0.62 (0.53–0.72)**  **p < 0.001** | **0.59 (0.51–0.70)**  **p < 0.001** |
| **Metro:** |  |  |
| Yes | *Ref* | *Ref* |
| No | 1.10 (0.91**–**1.34)  p = 0.315 | 1.14 (0.93**–**1.40)  p = 0.216 |
| **Age (years):** |  |  |
| 14 | *Ref* | *Ref* |
| 15 | 0.86 (0.69**–**1.08)  p = 0.195 | 0.86 (0.68**–**1.07)  p = 0.179 |
| 16 | **0.76 (0.61–0.97)**  **p = 0.025** | **0.75 (0.59–0.94)**  **p = 0.014** |
| 17 | **0.62 (0.50–0.78)**  **p < 0.001** | **0.61 (0.49–0.76)**  **p < 0.001** |

**Sports Participation**

1. **Sample characteristics and bivariate associations between demographic and diagnostic variables and sport participation status.**

**Table S3. Sample characteristics and bivariate associations between demographic and diagnostic variables and sport participation status in the past 12 months among U.S. youth aged 14–17 years.** Values are presented as unweighted n / weighted n (weighted column %). Estimates account for the complex sampling design of the National Survey of Children’s Health, including stratification, clustering, and sampling weights. Group differences were assessed using Rao–Scott adjusted chi-square tests.

|  | **All (N=26,308) n / weighted n (%)** | **No Sport (N=12,133) n / weighted n (%)** | **Yes Sport (N=14,175) n / weighted n (%)** | **Rao-Scott χ²; p-value** |
| --- | --- | --- | --- | --- |
| **Diagnosis:** |  |  |  |  |
| No ASD or ID | 25,024 / 11,394,819 (95.7%) | 11,139 / 5,527,976 (93.2%) | 13,885 / 5,866,843 (98.3%) | **χ² = 57.77; p = <0.001** |
| ASD only | 868 / 331,385 (2.8%) | 685 / 268,341 (4.5%) | 183 / 63,044 (1.1%) |  |
| ID only | 221 / 86,506 (0.7%) | 154 / 64,791 (1.1%) | 67 / 21,715 (0.4%) |  |
| ASD+ID | 195 / 88,739 (0.7%) | 155 / 70,993 (1.2%) | 40 / 17,746 (0.3%) |  |
| **Race:** |  |  |  |  |
| White | 20,318 / 8,455,577 (71.0%) | 9,095 / 4,115,244 (69.4%) | 11,223 / 4,340,333 (72.7%) | **χ² = 7.53; p = 0.006** |
| Not-White | 5,990 / 3,445,872 (29.0%) | 3,038 / 1,816,857 (30.6%) | 2,952 / 1,629,015 (27.3%) |  |
| **Poverty Ratio:** |  |  |  |  |
| >200% Poverty | 19,725 / 7,798,982 (65.5%) | 8,042 / 3,314,493 (55.9%) | 11,683 / 4,484,489 (75.1%) | **χ² = 205.15; p = <0.001** |
| ≤200% Poverty | 6,583 / 4,102,468 (34.5%) | 4,091 / 2,617,608 (44.1%) | 2,492 / 1,484,860 (24.9%) |  |
| **Sex:** |  |  |  |  |
| Male | 13,542 / 5,987,230 (50.3%) | 5,892 / 2,819,084 (47.5%) | 7,650 / 3,168,145 (53.1%) | **χ² = 18.42; p = <0.001** |
| Female | 12,766 / 5,914,219 (49.7%) | 6,241 / 3,113,016 (52.5%) | 6,525 / 2,801,203 (46.9%) |  |
| **Metropolitan Residence:** |  |  |  |  |
| Yes | 21,393 / 10,368,789 (87.1%) | 9,953 / 5,205,535 (87.8%) | 11,440 / 5,163,254 (86.5%) | χ² = 3.28; p = 0.070 |
| No | 4,915 / 1,532,661 (12.9%) | 2,180 / 726,566 (12.2%) | 2,735 / 806,095 (13.5%) |  |
| **Age (years):** |  |  |  |  |
| 14 | 5,862 / 3,072,471 (25.8%) | 2,396 / 1,398,992 (23.6%) | 3,466 / 1,673,479 (28.0%) | **χ² = 8.76; p = <0.001** |
| 15 | 6,427 / 3,027,145 (25.4%) | 2,805 / 1,465,535 (24.7%) | 3,622 / 1,561,610 (26.2%) |  |
| 16 | 6,964 / 2,947,343 (24.8%) | 3,305 / 1,512,635 (25.5%) | 3,659 / 1,434,708 (24.0%) |  |
| 17 | 7,055 / 2,854,490 (24.0%) | 3,627 / 1,554,938 (26.2%) | 3,428 / 1,299,552 (21.8%) |  |

1. **Crude and adjusted odds ratios for any sport participation**

**Table S4: Crude and adjusted odds ratios for any sport participation in the past 12 months.** Estimates are from survey-weighted logistic regression models accounting for complex survey design. Adjusted models include diagnosis, sex, race, poverty ratio, metropolitan status, and age. Reference groups are indicated.

|  | **Crude OR**  **(95% CI)**  **p-value** | **aOR**  **(95% CI)**  **p-value** |
| --- | --- | --- |
| **Diagnosis:** |  |  |
| No ASD or ID | *Ref* | *Ref* |
| ASD only | **0.22 (0.16–0.30)**  **p < 0.001** | **0.20 (0.15–0.27)**  **p < 0.001** |
| ID only | **0.32 (0.20–0.51)**  **p < 0.001** | **0.31 (0.19–0.51)**  **p < 0.001** |
| ASD+ID | **0.24 (0.14–0.41)**  **p < 0.001** | **0.24 (0.14–0.41)**  **p < 0.001** |
| **Race:** |  |  |
| White | *Ref* | *Ref* |
| Not-White | **0.85 (0.76–0.95)**  **p = 0.006** | **0.94 (0.83–1.07)**  **p = 0.332** |
| **Poverty Ratio:** |  |  |
| >200% Poverty | *Ref* | *Ref* |
| ≤200% Poverty | **0.42 (0.37–0.47)**  **p < 0.001** | **0.42 (0.37–0.47)**  **p < 0.001** |
| **Sex:** |  |  |
| Male | *Ref* | *Ref* |
| Female | **0.80 (0.72–0.89)**  **p < 0.001** | **0.77 (0.69–0.86)**  **p < 0.001** |
| **Metro:** |  |  |
| Yes | *Ref* | *Ref* |
| No | 1.12 (0.99–1.26)  p = 0.07 | **1.20 (1.06–1.37)**  **p = 0.005** |
| **Age (years):** |  |  |
| 14 | *Ref* | *Ref* |
| 15 | 0.89 (0.77**–**1.03)  p = 0.119 | 0.87 (0.75**–**1.01)  p = 0.073 |
| 16 | **0.79 (0.69–0.92)**  **p = 0.002** | **0.77 (0.66–0.89)**  **p < 0.001** |
| 17 | **0.70 (0.61–0.81)**  **p < 0.001** | **0.66 (0.57–0.77)**  **p < 0.001** |
